# Compensatory anabolic signaling in the sarcopenia of experimental chronic arthritis

**DOI:** 10.1038/s41598-017-06581-6

**Published:** 2017-07-24

**Authors:** Robert D. Little, Iván Prieto-Potin, Sandra Pérez-Baos, Amanda Villalvilla, Paula Gratal, Flavia Cicuttini, Raquel Largo, Gabriel Herrero-Beaumont

**Affiliations:** 1 Department of Epidemiology and Preventive Medicine, School of Public Health and Preventive Medicine, Monash University, Alfred Hospital, Melbourne, VIC 3004 Australia; 2 Bone and Joint Research Unit, Service of Rheumatology, IIS-Fundación Jiménez Díaz, Autonomous University of Madrid, Madrid, Spain; 3 Red Temática de Investigación Cooperativa de Envejecimiento y Fragilidad (RETICEF)-Instituto de Salud Carlos III, Madrid, Spain

## Abstract

Inflammatory activity in rheumatoid arthritis may alter the regulation of muscle mass leading to a secondary sarcopenia, commonly termed rheumatoid cachexia (RC). We characterized alterations to muscle structure and various pro-inflammatory, catabolic and regenerative markers in an animal model of RC. Antigen induced arthritis (AiA) was performed in 20 male adult rabbits. AiA animals exhibited significantly less weight gain, a markedly elevated serum C-reactive protein (CRP), lighter muscles with shorter cross-sectional diameter and increased myonuclei when compared to controls. Atrogin-1 and MuRF-1 were up-regulated alongside an increase in IL-1β, active NF-κB and a higher ratio of phosphorylated to inactive p38 MAPK. CCL-2 and TNF levels were reduced and IL-6 was unchanged between groups. We observed decreased pSTAT3, unchanged pSTAT1 and Myf5, but increased Pax7, MyoD and myogenin. AiA rabbits had a reduction in myostatin from gastrocnemii and synovium with a congruent decrease in serum myostatin compared to controls. Chronic arthritis induced an RC-like secondary sarcopenia with increased muscle protein breakdown. Elevated IL-1β may trigger proteolysis via elevated NF-κB and p38 MAPK signaling with a compensatory anabolic response suggested by myonuclear expansion, increased Pax7, MyoD and myogenin, reduced pSTAT3 as well as reduced serum, synovial and muscular myostatin.

## Introduction

Primary sarcopenia is a phenomenon of age-related loss of muscle mass and function affecting up to 30% of older adults^1^. Muscle loss in primary sarcopenia appears to be driven primarily by anabolic resistance. In contrast, secondary sarcopenia, such as the inflammatory muscle wasting of cancer, cardiac, and rheumatoid cachexia, appears to be driven by primarily catabolic processes. Additional pathways of muscle loss are activated by anorexia, asthenia and inactivity associated with chronic systemic inflammation^2^.

Rheumatoid cachexia (RC), the condition of reduced skeletal muscle mass with either stable or increased fat mass, affects between 11% and 26% of rheumatoid arthritis (RA) patients worldwide^3, 4^. The sarcopenia of RC has been linked to the increased risk of osteoporosis, metabolic syndrome and cardiovascular disease observed in RA^5, 6^. Muscle loss in RA has also been associated with weakness, imbalance and a reduced quality of life, independent of inflammatory severity and duration^7^.

Muscle homeostasis is maintained via a precise equilibrium between anabolic and catabolic processes. While poorly understood, inflammatory-mediated catabolism appears to be the major pathogenic factor in RC. Surrogate systemic markers of inflammation shown to correlate with altered body composition, including erythrocyte sedimentation rate (ESR)^8^, C-reactive protein (CRP)^9^, tumor necrosis factor alpha (TNF)^10^ and interleukin (IL)-6^11^. A range of anti- and pro-inflammatory cytokines are now known to be secreted from skeletal muscle^12^. Indeed, increased muscle-derived TNF has been demonstrated in previous experimental models of RC^13^. However, muscle-derived IL-1β, IL-6 and chemokine ligand 2 (CCL-2) under arthritic conditions have not been previously quantified. TNF, IL-6 and IL-1β activate the ubiquitin-proteasome system (UPS), a common proteolytic pathway^14^. The UPS can be activated via Nuclear Factor-kappa B (NF-κB) and p38 Mitogen-Activated Protein Kinases (MAPK) - two major intracellular signaling pathways in skeletal muscle^15^. UPS-mediated catabolism can be measured via the expression of muscle RING-finger protein-1 (MuRF-1) and atrogin-1, two muscle-specific E3 ubiquitin ligases, known as atrogenes^16^.

In contrast, the role of the UPS in primary sarcopenia is less pronounced, with multiple studies demonstrating no change or even decreased atrogene activity^17–19^. Instead, impaired anabolism appears to be the major source of muscle loss in primary sarcopenia. Unlike in RC, there is a large body of evidence demonstrating reduced testosterone, insulin-like growth factor 1, growth hormone and increased myostatin in age-related muscle loss^20^.

Myostatin is a negative growth factor primarily released from and acting on skeletal muscle^21^. The main mechanism of myostatin-induced wasting culminates in the suppression of anabolism, with an additional role in catabolic stimulation via the UPS^22, 23^. Myostatin is heavily implicated in primary and a number of secondary sarcopenic syndromes^24^. However, the exact role of myostatin and other muscle-derived messengers in RC is unclear.

Janus kinase (JAK)-mediated pathways are important intracellular mediators of muscle atrophy and regeneration^25^. Over 40 cytokines and growth factors signal via JAK-mediated pathways, including IL-6^26–28^. After receptor binding, JAKs become activated, triggering phosphorylation and stimulation of the signal transducer and activator of transcription (STAT) pathway^29^. Activation of various STAT genes has a complex regulatory role in muscle growth and repair^30^ – a topic not previously investigated in the secondary sarcopenia of chronic arthritis.

Adult skeletal muscle retains the ability to respond to injury. However, the anatomical and physiological skeletal muscle response following arthritic insult has not been extensively explored. Skeletal muscle regeneration occurs via activated satellite cells and is dependent on regulatory transcription factors, principally paired box 7 (Pax7)^31^. Other markers of muscle regeneration used to detect myocyte precursors include myogenic differentiation 1 (MyoD), to identify proliferating myoblasts, and myogenic factor 5 (Myf5) and myogenin to recognize differentiating myoblasts^32^.

We aimed to characterize the pattern of muscular injury and response in an animal model of chronic inflammatory arthritis. We evaluated alterations to muscle structure as well as the expression of various pro-inflammatory, catabolic and regenerative markers.

## Results

### Weight and CRP

**Figure 1:**
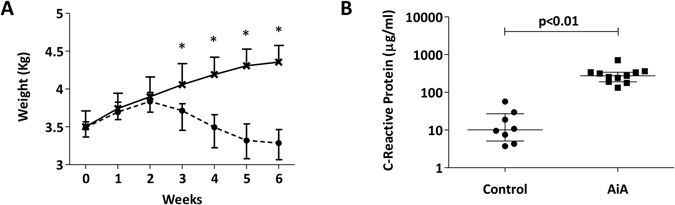
Effect of antigen-induced arthritis on weight and serum C-reactive protein (CRP). **(A**) Changes in body weight in AiA and control rabbits across the study, asterisks (*) denote statistically significant differences between AiA and control groups, p < 0.05. (**B**) Serum CRP levels (µg/ml) at the end of the study. Data represent individual values; medians with IQRs are also indicated (n = 8–11 rabbits per group).

The induction of AiA showed a reduction in rabbit weight in the AiA group compared to healthy controls from week 3 until euthanasia (Fig. 1A). Serum CRP was also increased in the AiA group compared to controls at the time of euthanasia (Fig. 1B).

### Structural skeletal muscle changes

**Figure 2:**
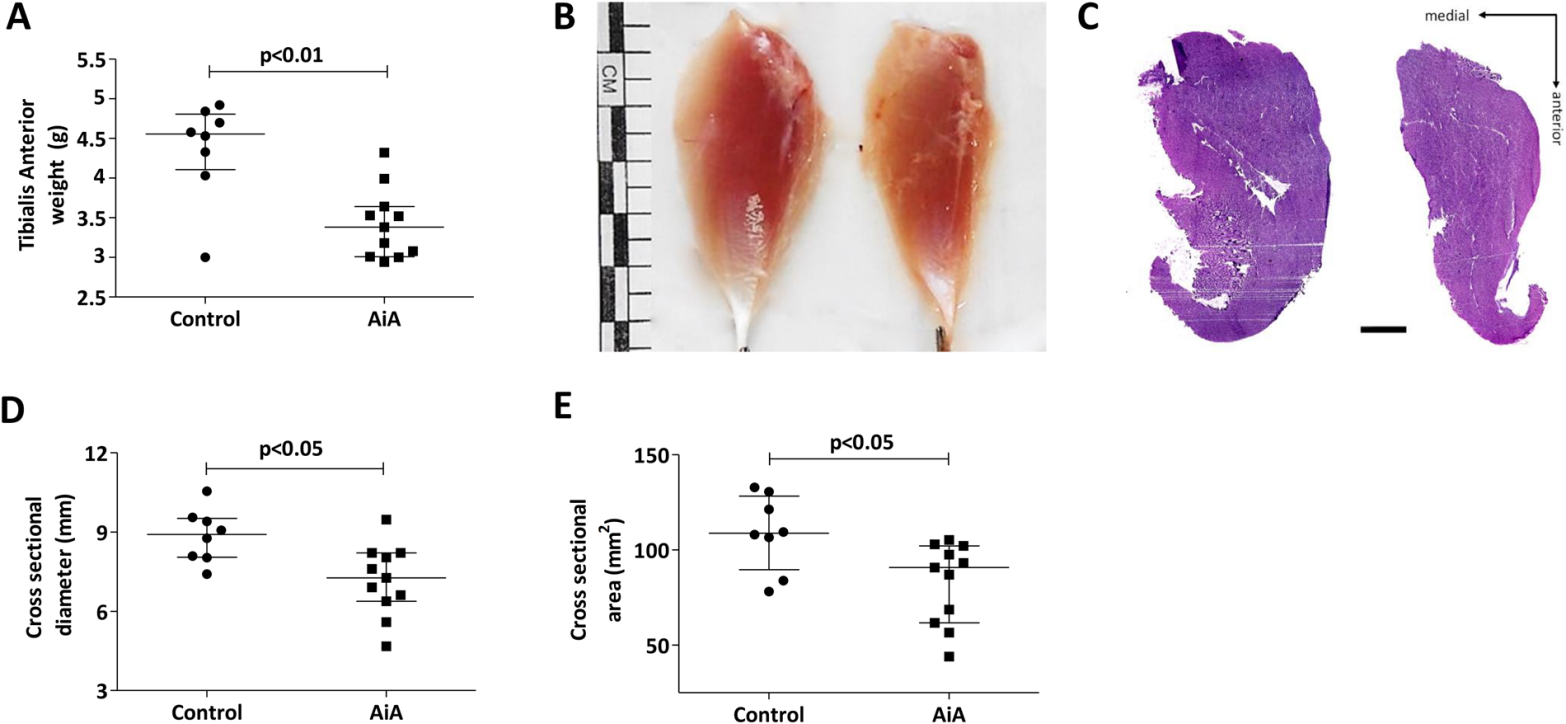
Alterations in skeletal muscle structure. (**A**) Tibialis Anterior (TA) weight (g). (**B**) Representative TA from healthy control (left) and antigen-induced arthritis (AiA) (right) groups illustrating reduced whole muscle size. Posterior view, scale bar = 1 cm. (**C**) Representative mid-belly cross-sections of TA in control (left) and AiA (right) groups stained with Harris haematoxylin and eosin. Scale bar = 2.5 mm. (**D**) Cross sectional diameter of TA (mm). (**E**) Cross sectional area of TA (mm^2^). Data represent individual values; medians with IQRs are also indicated (n = 8–11 rabbits per group).

AiA rabbits had reduced TA weight compared to controls, with a seemingly decreased size as demonstrated in posterior macroscopic photographs (Fig. 2A,B). Normalization of TA weight according to baseline body weight confirmed an independent significant difference between the groups (AiA 0.9 (0.9–1.1) vs. control 1.3 (1.2–1.4), p < 0.05). Measurement of hematoxylin and eosin stained sections confirmed gross muscle atrophy with reduced TA CSD and CSA in AiA rabbits compared to controls, as shown in Fig. 2C–E.

### Histological alterations

**Figure 3:**
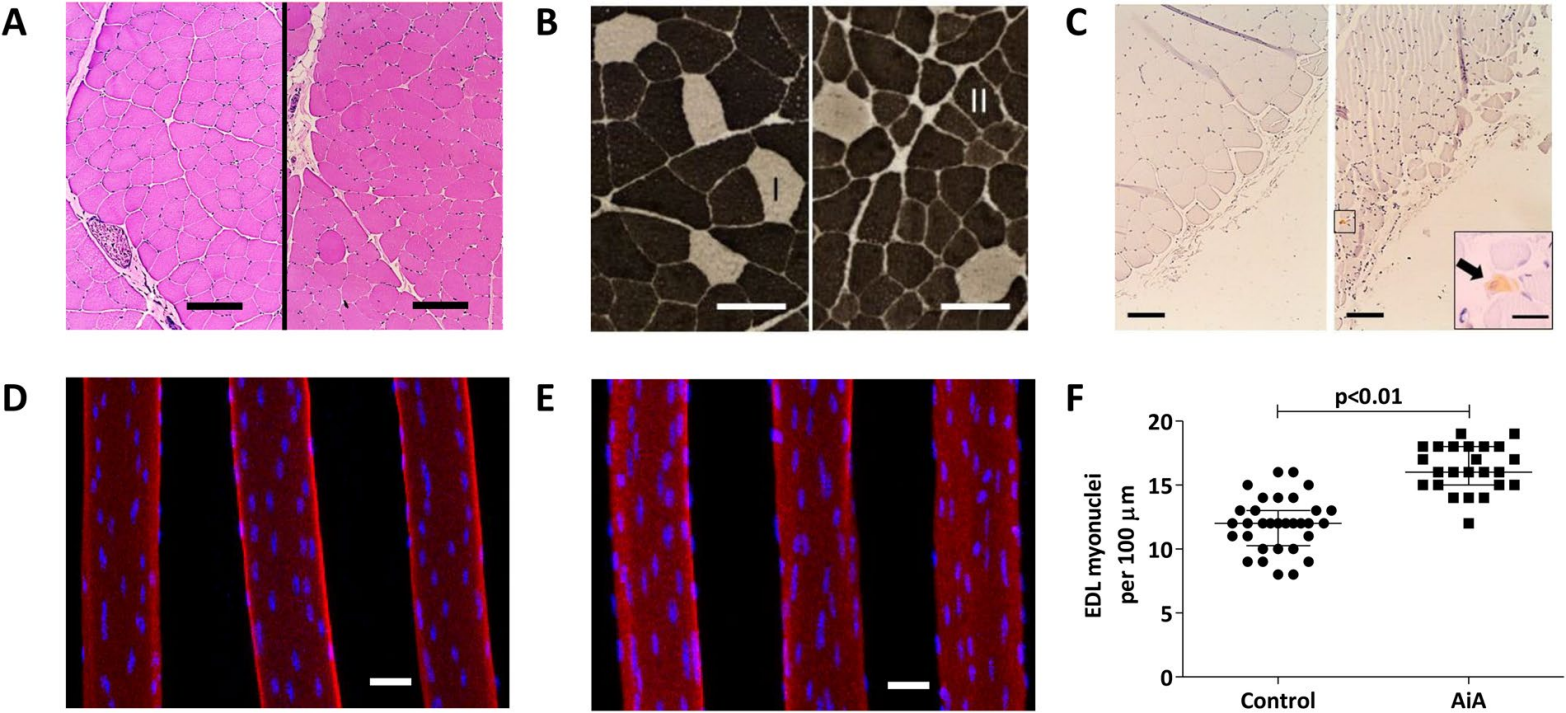
Histological alterations in skeletal muscle. (**A**) Representative sections of tibialis anterior (TA) of control (left) and AiA (right) rabbits. Carazzi haematoxylin and eosin staining. Scale bar = 100 μm. (**B**) Representative sections of type I (white) and II (black) fiber distribution and size in TA of control (left) and AiA (right) animals stained with ATPase pH 9.4. Scale bar = 100 μm. (**C**) Representative TA cross-section with RAM11 immunostaining of control (left) and AiA (right) groups. Scale bar = 100 μm. Inset showed RAM11 immunoreactive cell (black arrow) beneath the epimysium in AiA. Scale bar = 25 μm. (**D**–**F**) Representative focal microscopy images from segments of extensor digitorium longus (EDL) fibers in control (**D**) and AiA rabbits (**E**). Rhodamine phalloidin (red) was used to stain actin and DAPI (blue) to stain nuclei. Scale bar = 30 µm. (**F**) Myonuclei number in control in comparison to AiA group expressed as number of nuclei per 100 mm of fiber segment.

Qualitative observation of hematoxylin and eosin cross-sections suggests a heterogeneous fiber size and distinct nuclei number in the AiA group in comparison to muscle fibers from controls (Fig. 3A). In addition, ATPase staining illustrates a gross reduction in type II fiber size in AiA rabbits (Fig. 3B).

After describing qualitative skeletal muscle changes, we aimed to study whether systemic inflammation would lead to greater inflammatory cell infiltrate within skeletal muscle. For that purpose we characterized the presence of RAM11 positive macrophages. A small population of positive RAM11 cells were present in 60% of TA cross-sections from the AiA group. However, macrophages appeared to be localized deep to the epimysium with relative endomysial sparing (Fig. 3C). None of the sections from the control group demonstrated RAM11 positive cells.

Rhodamine-phalloidin and DAPI staining of EDL revealed distinct shape and a different spatial organization of nuclei (Fig. 3D,E) together with a significant increase in myonuclear number in AiA rabbits compared to healthy controls (Fig. 3F).

### Activation and differentiation markers in muscle regeneration

**Figure 4:**
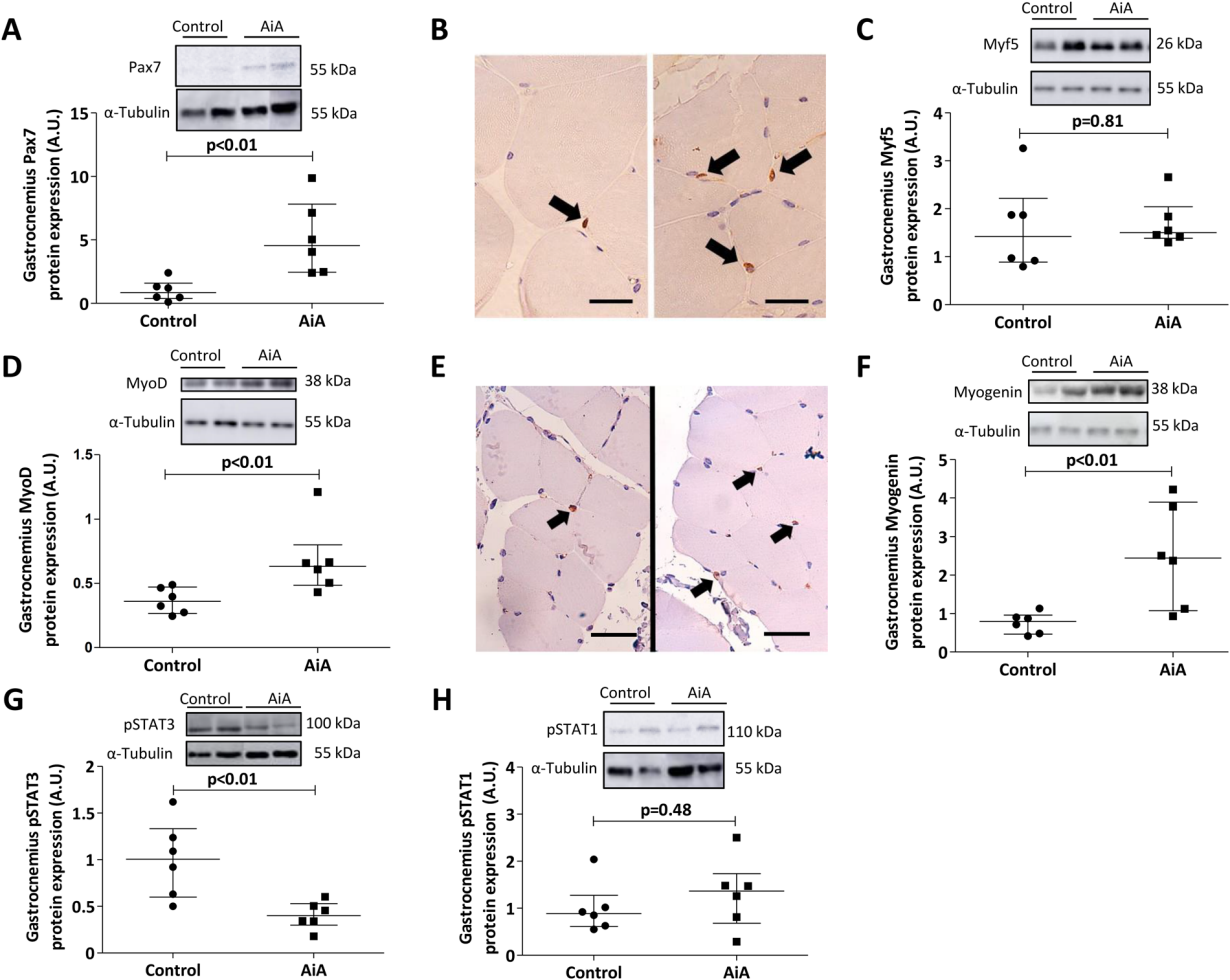
Activation and differentiation markers in muscle regeneration. Densitometric analysis of Pax7 (**A**), Myf5 (**C**), MyoD (**D**), Myogenin (**F**), pSTAT3 (**G**) and pSTAT1 (**H**) protein expression levels in gastrocnemius. Data are normalized to endogenous control (α-tubulin) and expressed as arbitrary units (A.U.). Representative cropped blots of two animals of each group are shown, control and AiA, respectively. Full length blots are presented in Supplementary Figures 1 and 2. Data represent individual values and medians with IQRs are also indicated (n = 6 rabbits per group). Representative TA cross sections with Pax7 (**B**) and MyoD (**E**) immunoreactive nuclei (black arrows) in control (left) and AiA (right) groups. Scale bar = 25 μm. Pax7 = paired box protein 7, Myf5 = myogenic factor 5, MyoD = myogenic differentiation 1, pSTAT = phosphorylated signal transducer and activator of transcription.

We then studied whether the increased cell number in AiA muscle sections was related to an increase in the satellite cell population. The AiA group showed increased Pax7 protein expression in the gastrocnemii of AiA rabbits compared to controls (Fig. 4A). This finding is supported by greater immunoreactivity for Pax7 on histological sections (Fig. 4B).

In order to assess the activation and differentiation of myogenic cells in the gastrocnemii of our animals, we explored the protein expression of Myf5, MyoD and myogenin. Whereas Myf-5 protein levels remained unchanged (Fig. 4C), MyoD and myogenin levels were elevated in the AiA group (Fig. 4D,F). MyoD immunoreactivity confirmed the protein expression results (Fig. 4E).

We also studied the activation of STAT genes to analyze their behavior following catabolic insult. STAT3 phosphorylation was significantly reduced in the gastrocnemii of AiA rabbits compared to controls, whereas STAT1 phosphorylation remained unchanged (Fig. 4G,H).

### Pro-inflammatory mediators and atrogenes

**Figure 5:**
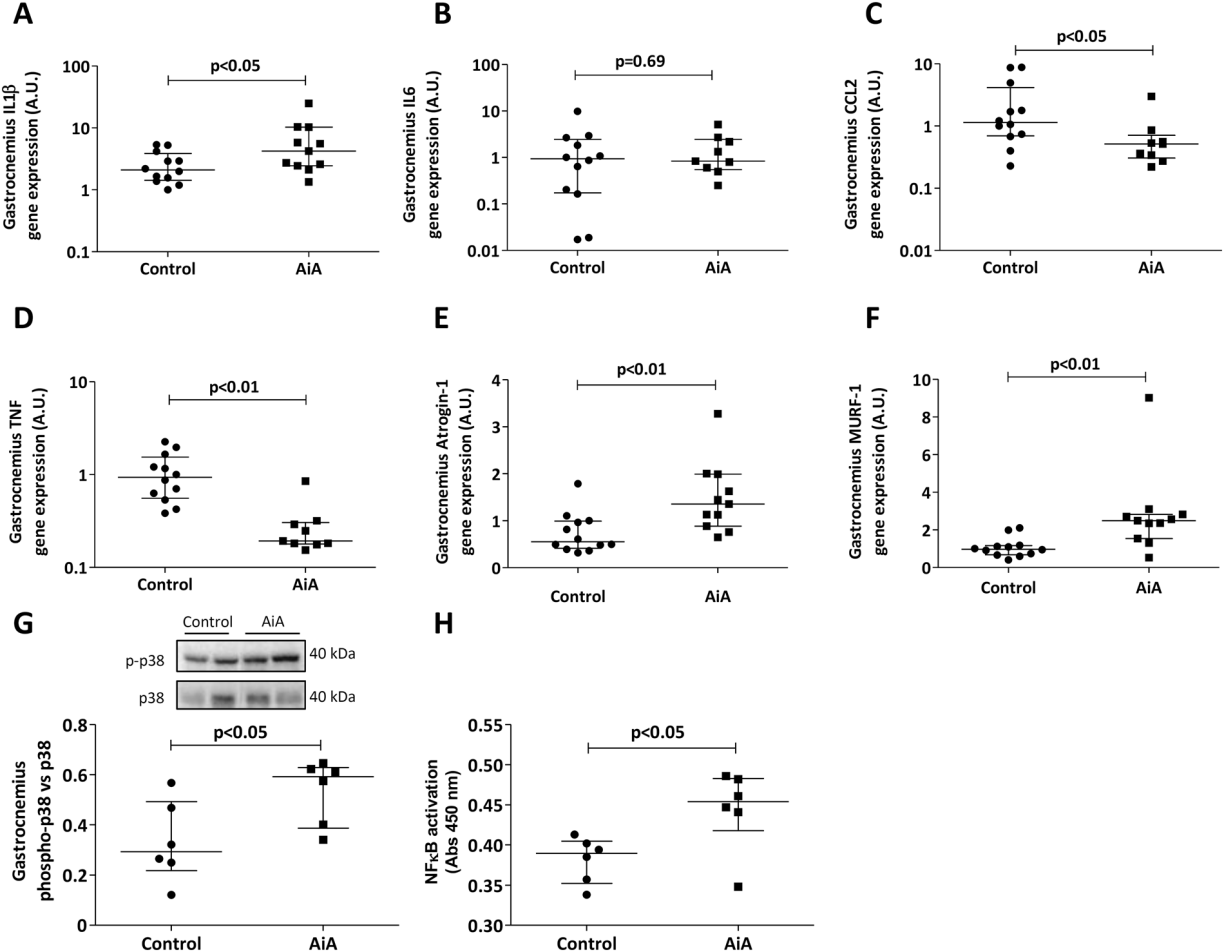
Pro-inflammatory mediators and atrogenes. Gene expression of control and AiA rabbits normalized to endogenous control in gastrocnemius. Data represent individual values and medians with IQRs are also indicated (n = 6 rabbits per group, both limbs of each rabbit are represented) for IL-1β (**A**), IL-6 (**B**), CCL-2 (**C**), TNF (**D**), atrogin-1 (**E**) and MuRF1 (**F**). (**G**) Protein expression of p38 MAPK. Data are expressed as arbitrary densitometric units (A.U.) and normalized relative to the expression of phosphorylated p38 MAPK. Representative cropped blot of two animals of each group are shown, control and AiA, respectively. Full-length blot is presented in Supplementary Figure 2. (**H**) Active protein levels of NF-κB measured by enzyme linked immunoabsorbant assay. Graphs show individual values and medians with IQRs are also indicated (n = 6 rabbits per group). IL = interleukin, CCL-2 = C-C Motif Chemokine Ligand 2, TNF = tumor necrosis factor, MuRF1 = muscle RING-finger protein-1.

Gene expression of IL-1β was increased in the gastrocnemii of AiA compared to controls (Fig. 5A). However, expression of IL-6 was equivalent between the two groups (Fig. 5B) and both CCL-2 and TNF were significantly reduced in AiA rabbits compared to controls (Fig. 5C,D).

In regards to atrogene expression, there was a significant up-regulation in both atrogin-1 and MuRF-1 in AiA rabbits compared to controls (Fig. 5E,F).

In order to investigate whether IL-1β could be responsible for the increase in atrogin-1 and MuRF-1, we measured the activity of two downstream intracellular pathways known to stimulate atrogene expression –p38 MAPK and NF-κB. We found a significant increment in the ratio of phosphorylated p38 MAPK/p38 MAPK in AiA animals (Fig. 5G), along with higher levels of active NF-κB able to bind its consensus sequence within the nucleus (Fig. 5H).

### Local and systemic myostatin levels

**Figure 6:**
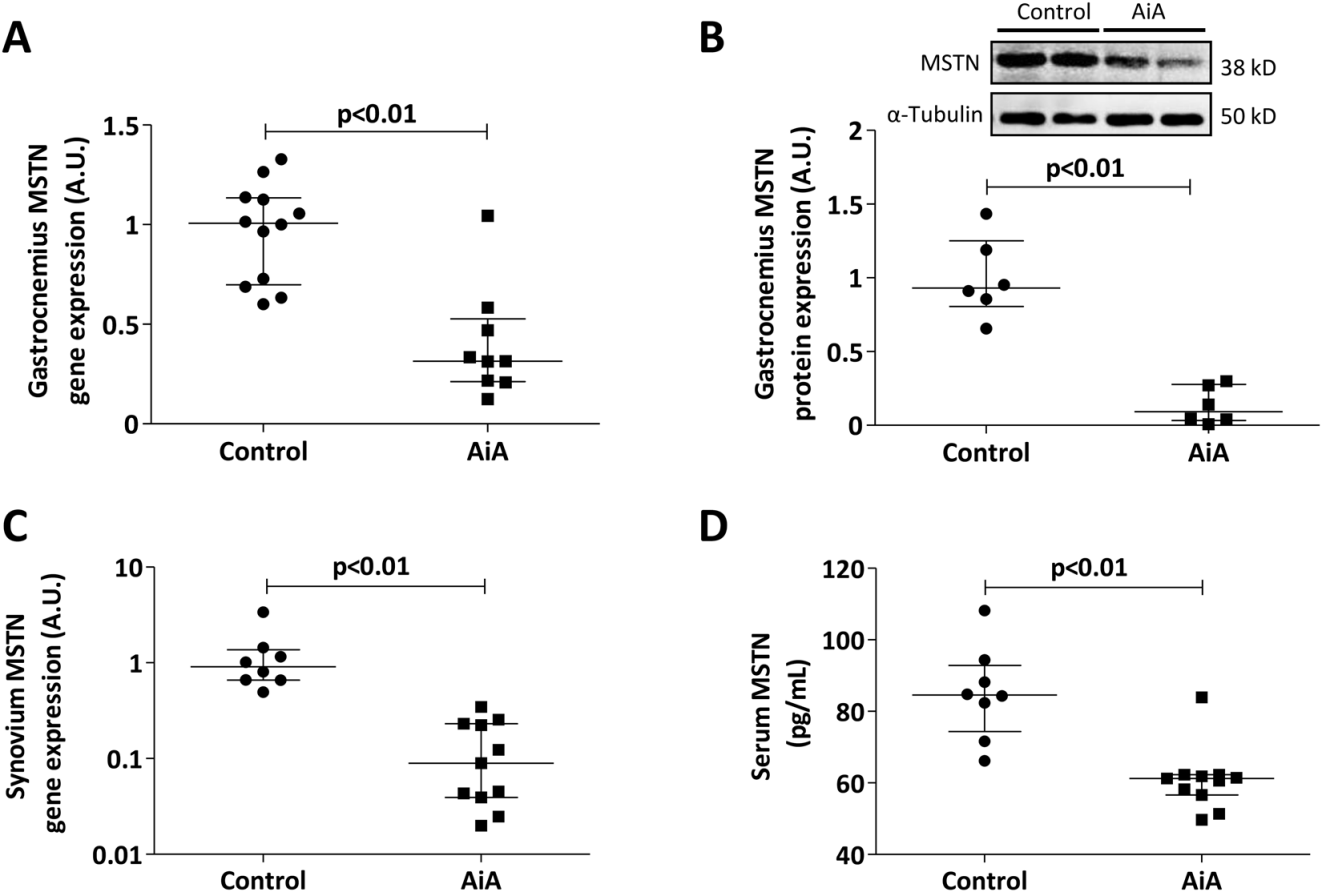
Local and systemic myostatin levels in skeletal muscle, synovium and serum. (**A**) Myostatin gene expression in gastrocnemius from control and AiA rabbits (n = 6 rabbits per group, both limbs of each rabbit are represented). (**B**) Myostatin protein expression in gastrocnemius from control and AiA rabbits. Data for protein expression are expressed as arbitrary densitometric units (A.U.) and normalized relative to the expression of α-tubulin (n = 6 rabbits per group). Representative cropped blot of two animals of each group are shown, control and AiA, respectively. Full length blot is presented in Supplementary Figure 1. (**C**) Myostatin gene expression in the synovium. (**D**) Serum myostatin levels in control and AiA rabbits. MSTN = Myostatin. Graphs show individual values and medians with IQRs are also indicated (n = 8–11 rabbits per group).

Finally, we studied the role of myostatin as an anabolic mediator in systemic inflammation. We found a significant reduction in both mRNA and protein expression of myostatin from gastrocnemii of AiA rabbits (Fig. 6A,B). Both synovial membrane and serum myostatin expression were also reduced in AiA animals when compared to controls (Fig. 6C,D).

## Discussion

We have studied the histological alterations of muscle wasting in an animal model of chronic arthritis and the derangement of various markers of catabolism and regeneration. We observed an elevated serum CRP together with a significant decrease in body weight and muscle size. While RC in humans is widely reported to be associated with stable weight or weight gain, our results are consistent with previous animal models of inflammatory arthritis in rabbits^33^, rats^34^, mice^35^ and monkeys^36^. Similarly, our findings of reduced muscle CSA, CSD and weight is reflected in previous models across a range of species^36–38^. The loss in muscle size and weight, and the heterogenous myofibre size observed in AiA rabbits may reflect our qualitative observation of preferential wasting of type II muscle fibres, an established phenomenon described in experimental arthritis in other species^36^.

Elevated muscle-derived IL-1β may have contributed to the observed atrophy, structural derangement and increased atrogene expression in arthritic rabbits. Receptors for IL-1β have been found on skeletal muscle cells^39^ and the administration of IL-1β has been shown to stimulate MuRF-1 and atrogin-1 mRNA expression with subsequent atrophy *in vitro* ^15^. The modest up-regulation of NF-κB activation and elevated ratio of phosphorylated p38 MAPK/p38 MAPK protein in arthritic rabbits may reflect the intracellular effector pathways by which IL-1β contributes to proteolysis. IL-1β has been shown to stimulate NF-κB and p38 MAPK signaling with subsequent atrogene induction and myotube atrophy *in vitro* ^15, 40, 41^. Therefore, we suggest that the increased atrogin-1 and MuRF-1 expression as well as a proportion of the sarcopenia observed in AiA rabbits may have been driven by direct autocrine IL-1β signaling.

According to our data, animals with AiA displayed signs of simultaneous muscle wasting and repair. The myonuclear expansion observed in arthritic rabbits is an anatomical marker of muscle growth and regeneration^42^. Indeed, myonuclei number has been shown to increase after sarcopenic challenge in rats^43^. Increased Pax7 protein expression alongside greater Pax7 immunoreactivity in AiA rabbits suggests that satellite cell proliferation is responsible for a proportion of the increased myonuclear mass. In the presence of myogenic regulatory factors, Pax7-expressing satellite cells have the ability to self-renew, differentiate and fuse in order to repair injured skeletal muscle^44^. In line with experimental arthritis in other species^13, 45^, our model of AiA demonstrated elevated MyoD and myogenin, although contradictory results have been published^34^. Increased MyoD enables satellite cell proliferation and up-regulation of myogenin thereby facilitating myoblast differentiation and muscle regeneration^32, 46^. Our finding of equivalent Myf5 protein expression between groups may reflect the non-obligatory and transient expression of Myf5 in adult myogenesis^47^.

We propose that the reduced myostatin and pSTAT3 expression from AiA rabbits may contribute to the observed myonuclear expansion and increased Pax7^30, 48^. In addition to promoting cellular and particularly satellite cell expansion, the decreased pSTAT3 expression in the AiA group may have ameliorated UPS-mediated proteolysis. While IL-6 mRNA was equivalent in both groups, decreased expression of its downstream intracellular effector may decrease atrogin-1 expression and down-regulate the acute phase response, thereby addressing the proportion of sarcopenia that is driven by inflammation^25, 49^.

Perhaps the most striking marker of anabolic compensation in our model is reduced myostatin expression across multiple sites. Myostatin negatively regulates multiple aspects of skeletal muscle homeostasis, primarily via decreased myogenesis^22, 23, 50^. Despite involvement in the muscular wasting associated with cancer^51^, congestive heart failure^52^ and chronic obstructive pulmonary disease^53^, a role for myostatin in RC has not been consistently demonstrated. While Dankbar *et al*. recently demonstrated elevated myostatin expression in synovial membranes of patients with RA, it is not known whether these patients were also affected by RC^54^. Furthermore, of the seven previous models of inflammatory arthritis that we found in the literature, only three reported increased myostatin mRNA or protein^36, 45, 54^. A number of the conflicting reports may originate from myostatin mRNA-protein discordance. However, concordance between myostatin mRNA and protein expression from skeletal muscle in our model suggests a true reduction in myostatin secretion.

An alternative explanation for our results may be found in our methodology - including variance in species, method of arthritis induction and the model duration. Ramirez *et al*. found a 4.3-fold increase in myostatin mRNA at two days, which decreased to a 2-fold increase at seven days and then returned to baseline 15 days post-arthritis induction^45^. These data may suggest a role for myostatin in early RC-induced sarcopenia that diminishes over a prolonged period. We propose that the extended duration of our model may enable a compensatory down-regulation of myostatin with a subsequent anabolic stimulus. Such myostatin suppression has been observed in regenerating muscles in mice models of muscular dystrophy^55^. Furthermore, a recent study of postoperative patients found significantly reduced serum myostatin at two postoperative time points, with the lowest myostatin levels coinciding with the highest serum CRP^56^. The relationship between inflammatory atrophy and compensatory myostatin inhibition requires further investigation to determine its merit and the mechanism of down-regulation.

Finally, we found a reduction in TNF mRNA expression in arthritic animals. While induction of the UPS after exposure to exogenous TNF has been previously demonstrated^57^, this observation is not universal^58, 59^. In addition, despite previous animal models demonstrating increased TNF expression in RC^13, 37^, its role in humans is less conclusive. Previous studies of anti-TNF agents in RA patients have failed to show significant improvements in fat-free mass^60–63^. Further research is clearly indicated to determine the exact role of TNF in the sarcopenia of inflammatory arthritis. In a similar pattern to TNF, CCL-2 was also reduced in AiA rabbits. CCL-2 and subsequent macrophage attraction in acute inflammation is thought to be critical for future muscle repair^64^. However, the decreased CCL-2 expression seen in the arthritis group may be a reflection of the chronicity of our model. Indeed, the finding of sub-epimysial RAM11 positive cells may indicate prior CCL-2-driven macrophage infiltration.

A limitation of our study is the lack of a precise distinction between the effect of arthritis-induced systemic inflammation from that of pain-induced inactivity. Whereas disuse atrophy is a relatively uncomplicated form of muscle loss, mostly dependent on the loss of a mechanical input, in inflammatory disease states such as cancer, HIV/AIDS, sepsis and rheumatoid arthritis, there is also a meaningful involvement of a complex pro-catabolic, hormonal and cytokine environment to contemplate. In these conditions, systemic inflammation is often associated with asthenia, anorexia and inactivity due to pain^2^. Each factor contributes to the overall atrophy via a complex interplay between the various pathways of muscle loss. Therefore, even when employing controlled experimental models, it is difficult to isolate the specific contribution of each component. It is very likely that an undetermined contribution of inactivity due to pain and discomfort may exist in the muscle alterations described in this study, as it happens in RA patients^65^. Although previous experimental models of muscle disuse atrophy showed increased atrogene expression with an associated transient increase in local inflammatory response, arthritis does appear to cause an independent and distinct pattern of muscle wasting in the direct comparison trials of disuse and arthritic atrophy^16, 34, 45^. Furthermore, our data suggest a systemic sarcopenic effect as showed by the similar extent of lower limb muscles and total body weight loss in AiA.

Our study has confirmed the muscular atrophy of RC in a new species. After investigating different pathogenic processes, we suggest that chronic arthritis alters the precise homeostatic equilibrium of skeletal muscle remodeling. The systemic proinflammatory milieu and local elevated IL-1β may activate two major intracellular pathways of proteolysis. In addition, we have presented anatomical, mRNA and protein markers of compensatory anabolic activity. Myonuclear expansion, down-regulation of myostatin signaling, increased MyoD and myogenin and decreased pSTAT3 signaling may all reflect attempts to repair the catabolic insult of inflammatory arthritis. Subsequent research is required to evaluate these hypotheses and convert them into muscle-specific therapies that may allow maintenance of skeletal muscle mass in patients suffering from RC.

## Methods

### Experimental model in rabbits

Adult, male New Zealand rabbits with a body weight of 3 to 3.5 kg (Granja San Bernardo, Navarra, Spain) were used for the experiment. Animal handling and experimentation were performed in accordance with Spanish Regulations and the Guidelines for the Care and Use of Laboratory Animals drawn up by the National Institutes of Health (Bethesda, MD, USA). The experimental protocol was approved by the Institutional Ethics and Welfare Committee of the Institute and Health Research Jiménez Díaz Foundation.

After two weeks of adaptation to our facilities, 20 rabbits were randomly assigned into two groups – antigen-induced arthritis (AIA) and control. Arthritis was induced according to a previously described protocol^66^. Briefly, animals were given two intradermal injections of 1 ml ovalbumin (OVA) (4 mg/ml; Sigma-Aldrich, St. Louis, MO, USA) in Freund’s complete adjuvant (Difco, Detroit, MI, USA). Five days after the second injection, 1 ml of OVA (5 mg/ml in 0.9% NaCl) was injected intra-articularly into the knee joints on a weekly basis over the following four weeks. One AIA animal died of unknown cause and the remaining rabbits completed the study (n = 11). Control rabbits underwent no experimental intervention (n = 8).

### Tissue collection

At the end of the study, rabbits were bled from their marginal ear vein, and then euthanized with an overdose of intra-cardiac sodium thiopental (50 mg/kg; Tiobarbital, Braun Medical SA, Barcelona, Spain). The left lower limb was shaved and an anterior skin incision was made from patella to mid-paw to dissect tibialis anterior (TA) and extensor digitorum longus (EDL). Proximal gastrocnemius and synovia from both limbs were also collected in six animals from each group for gene and protein expression studies, snap frozen in liquid nitrogen and stored at −80 °C.

Post-dissection, TA was immediately transferred to a cell plate for anterior and posterior photographs. A 1mm segment of the TA mid-belly, defined as the widest identifiable region, was sectioned, dehydrated in talc powder and placed cross-sectionally in a template of Optimal Cutting Temperature compound (OCT; Fisher Scientific, USA). Each OCT embedded cross-section was then set in liquid nitrogen before storage at −80 °C for sectioning, staining and measurement. The distal portion of the remaining TA was fixed in formaldehyde 4% and then paraffin embedded for detection of RAM11, Pax7 and MyoD immunoreactivity.

EDL muscles were transferred to 0.2% collagenase type I (Sigma-Aldrich, St Louis, Mo, USA) in Dulbecco’s modified eagle’s medium (DMEM, Lonza, Basel, Switzerland), 4.5 g/L glucose, 1% L-glutamine with 110 mg/ml sodium pyruvate, and incubated at 37 °C for 1 hour. Under a stereoscopic microscope, EDL muscle was flushed in warm medium until fibers were released, as previously described^67^.

### Serum biochemical markers

Ten milliliters of blood were used for serum extraction. Specific enzyme-linked immunosorbent assay kits were used to measure CRP (Abcam, ab157726, Cambridge, UK) and myostatin (Cloud Clone Corp., CEB653, Houston, TX, USA).

### Histological studies

Frozen TA muscles were sectioned at −30 °C in consecutive 5 μm slices with a Leica CM1850 cryostat (Leica Co., Germany). Two consecutive sections were oriented in the same plane then attached to a single microscope slide (Superfrost PLUS; Thermo Scientific, USA).

Hematoxylin and eosin stained TA cross-sections were photographed using an automated iScan Coreo slide scanner (Ventana Medical Systems, USA) with a maximum objective of 20x. The cross-sectional diameter (CSD) and cross-sectional area (CSA) were measured using the Image Viewer software package (Ventana Medical Systems, USA). CSD was measured using the Feret’s diameter, defined as the maximum diameter across the lesser aspect of the section. Parallel horizontal lines at the top and bottom of the image ensured consistent perpendicular measurements. CSA was calculated automatically after tracing the outline of the cross-section. All measurements were taken manually by two observers blinded to each group.

ATPase staining was performed in TA cross-sections. Briefly, cross-sections were incubated 45 minutes in 0.1 M glycine/NaCl buffer adjusted to pH 9,4 with 0,75 M CaCl2 and 5 mg ATP (Sigma-Aldrich, St Louis, Mo, USA), rinsed in distilled water and afterwards incubated in 2% cobalt chloride, then immersed in dilute (1/10) ammonium chloride to develop type 1 (white) and type 2 fiber (black) delineation, and finally dehydrated in ascending alcohol series and mounted with di-n-butylphthalate in xylene (DPX, mounting medium, VWR International Ltd., Lutterworth, UK).

### Immunohistochemistry

Paraffin embedded TA was sectioned at a width of 2 microns. Macrophages were visualized using mouse monoclonal RAM11 primary antibody (36.2 mg/L at a 1/100 dilution; Dako, M0633, Golstrup, Denmark), satellite cells were immunoreactive to Pax 7 (28 μg/ml; Developmental Studies Hybridoma Bank, AB 528428, Iowa City, IA, USA) and MyoD (13.5 μg/ml; Dako, M3512, Carpinteria, CA, USA). 10 mM citrate buffer was used for antigen retrieval (pH 6, 30 minutes at 85 °C). Primary antibodies were detected with sheep anti-mouse (Amersham, RPN1001, Arlington Heights, IL, USA) and anti-goat (Merck Millipore, AB324P, Darmstadt, Germany) IgG secondary antibodies at 1/100 and 1/250 dilution and visualized with a horseradish peroxidase/ABC complex using 3,3 diaminobenzidine tetra-hydrochloride as the chromogen (Dako, K3468, Golstrup, Denmark). Sections were counterstained with hematoxylin and mounted with DPX medium. Photographs were obtained using a Leica DMD108 digital micro-imaging instrument (Leica, Microsystems, Inc. Buffalo Grove, IL, USA) at 10x and 40x magnification.

### Fluorescent labeling of EDL fibers

Ten fiber segments from each animal were collected in a plate covered with matrigel (Corning, NY, USA) forming two different pools, AiA and control. From each pool, fiber segments were transferred to a chamber slide and fixed in 4% formaldehyde, then rinsed and incubated in 0.5% Triton to allow permeabilization. Fibers were incubated in the dark in 100 nM rhodamin-phalloidin (Cytoskeleton, Denver, CO, USA) over 45 minutes. DAPI (1 μg/ml﻿, Invitrogen, Eugene, OR, USA) was used to counterstain nuclei during 30 minutes at room temperature. Finally, slides were mounted in Fluorsave (Calbiochem, Merck Millipore, Darmstadt, Germany) and visualized through Leica TCS SP2 instrument (Leica, Microsystems, Inc., Buffalo Grove, IL, USA). Nuclei count was performed using LAS AF software (Leica, Microsystems, Inc. Buffalo Grove, IL, USA) and expressed as number of nuclei per 100 μm of segment fiber.

### Gene expression

Total RNA was isolated from gastrocnemius and synovium using TriPure isolation reagent (Roche Diagnostics, Indianapolis, IN, USA), according to the manufacturer’s instructions. A total of 1 µg RNA was reverse-transcribed with a high capacity cDNA kit (Applied Biosystems, San Francisco, CA, USA) and RNA expression was quantified by single-reporter real time PCR using the StepOnePlus™ detection system and StepOne™ software v2.2 (Applied Biosystems, San Francisco, CA, USA). TaqMan® primers and probes were used to measure IL-6 (Oc04097053_m1), TNF (Oc03397715_m1), myostatin (Oc03399520_m1), IL-1 (Oc03823250_s1) and CCL-2 (Oc03823583_s1). Glyceraldehyde-3-phosphate dehydrogenase (GAPDH; Oc03823402_g1) was used as an endogenous control. SYBR Green® primers were designed using Primer3Plus and Primer-BLAST software from sequences obtained from GenBank (NIH, USA) and used to measure MuRF-1 (XM_008265917.1; Fw: CACCTTCCTCATGAGTGCCA, Rv: TCTGTCCCAAAGTCGATGGC), atrogin-1 (XM_002710762.2; Fw: TACTGCACTTTGGGGGAAGC, Rv: ATCAGTTCCAACAGCCGGAC) and the endogenous control, peptidylprolyl isomerase A (PPIA, NM_001082057.1; Fw: AGGGTTTATGTGCCAGGGTG, Rv: AAGATGVCCAGGACCTGTGTG). All target genes were normalized relative to the expression of the endogenous controls.

### Preparation of total and nuclear extracts from tissue

Frozen gastrocnemius was crushed using a mortar and a pestle cooled with liquid nitrogen. For the extraction of total protein, 50 mg of pulverized tissue was homogenized in a buffer containing 15 mM HEPES, 10% glycerol, 0.5% NP-40, 250 mM NaCl, 1 mM EDTA, 1 mM phenylmethanesulfonylfluoride (PMSF) and a phosphatase- and protease-inhibitor cocktail (Sigma-Aldrich, St. Louis, Mo, USA). Extracts were incubated 15 minutes on ice and centrifuged 15 minutes at 12 000 rpm and 4 °C. Supernatants were collected and protein concentration was determined by the BCA method.

For the preparation of nuclear extracts, 50 mg of pulverized frozen tissue was homogenized with a Dounce tissue grinder in a hypotonic buffer containing 20 mM HEPES, 5 nM NaF, 10 μM Na2MoO4, 0.1 mM EDTA, 0.01% NP-40, 1 M dithiotreitol (DTT) and a phosphatase- and protease-inhibitor cocktail (Sigma-Aldrich, St. Louis, Mo, USA). Extracts were centrifuged 10 minutes at 850 g and 4 °C. Cell pellets were gently re-suspended in hypotonic buffer, incubated 15 minutes on ice and centrifuged 30 seconds at 14 000 rpm and 4 °C. Nuclear pellets were then re-suspended in 50 μl of Complete Lysis Buffer (Active Motif, La Hulpe, Belgium), incubated 30 minutes on ice in a rocking platform and centrifuged 10 minutes at 14 000 rpm and 4 °C. Supernatants were collected and protein concentration was determined by the Bradford method.

### Western Blot

20 μg of total protein was resolved on 12% or 15% acrylamide - SDS gels and transferred to a nitrocellulose membrane in 48 mM Tris, 39 mM glycine and 20% methanol buffer at 25 V for 30 minutes at room temperature. Membranes were blocked in 5% skimmed milk in TBS Tween® 1 hour at room temperature and incubated overnight at 4 °C using anti-myostatin/GDF8 antibody (0.002 mg/ml at a 1/500 dilution; R&D Systems, AF788, Minneapolis, MN, USA), phospho-STAT3 (0.5 mg/ml at a 1/200 dilution; R&D Systems, MAB4607, Minneapolis, MN, USA), phospho-STAT1 (0.5 mg/ml at a 1/100 dilution; Affymetrix eBioscience, 14-9008, SanDiego, CA, USA) Pax7 (28 ug/ml, at 1/10 dilution, Developmental Studies Hybridoma Bank, AB 528428, Iowa City, IA, USA), p38 MAPK (1 mg/ml at a 1/1000 dilution; Millipore, ABS29, Temecula, CA, USA), phospho-p38 MAPK (1 mg/ml at a 1/1000 dilution; Abcam, ab45381, Cambridge, UK), Myogenin (1 mg/ml at a 1/500 dilution; Abcam, ab82843, Cambridge, UK) and MyoD (1 mg/ml at a 1/1000 dilution; Abcam, ab16148, Cambridge, UK). Antibody binding was detected by enhanced chemoluminescence using peroxidase-labeled anti-goat (Merck Millipore, Darmstadt, Germany), anti-rat (Thermo Scientific, Waltham, MA, USA) and anti-mouse (GE Healthcare LifeSciences Piscataway, NJ, USA) secondary antibodies at a 1/5000, 1/20000 and 1/5000 dilution, respectively. Expression levels of loading control were validated with anti-α-tubulin (Sigma-Aldrich, St Louis, Mo, USA) at 1/8000 dilution. Results were expressed as arbitrary densitometric units (A.U.) and normalized relative to the expression of α-tubulin.

### Measurement of NF-κB activation by enzyme linked immunosorbent assay (ELISA)

5 μg of nuclear protein were analyzed with the TransAM NF-κB p65 ELISA kit (#40096, Active Motif, La Hulpe, Belgium), according to the manufacturer’s instructions. Absorbance was measured at 450 nm and the relative amount of p65 NF-κB protein between control and AiA rabbits was calculated.

### Statistical analysis

All statistical analyses were performed using SPSS version 21.0 software for Windows (IBM, New York, NY, USA). Descriptive data are expressed as the median ± interquartile range (IQR). Comparisons between multiple groups used Kruskal-Wallis tests with Bonferroni correction of post hoc Mann–Whitney U tests, p < 0.05 was considered significant.

## Electronic supplementary material

Supplementary Information
