# Supplementary material for: Compensatory anabolic signaling in the sarcopenia of experimental chronic arthritis
Source: Sci Rep. 2017 Jul 24;7:6311. doi: 10.1038/s41598-017-06581-6 (PMC5524910; doi:10.1038/s41598-017-06581-6)
Supplement: Supplementary file 1 — Supplementary Information [file 41598_2017_6581_MOESM1_ESM.pdf]

Compensatory anabolic signaling in the sarcopenia of experimental chronic arthritis.

Robert D. Little[1], Iván Prieto-Potin[2,3], Sandra Pérez-Baos[2], Amanda Villalvilla[2], Paula Gratal[2], Flavia Cicuttini[1], Raquel Largo[2,3], Gabriel Herrero-Beaumont[2,3]

[1]Department of Epidemiology and Preventive Medicine, School of Public Health and Preventive Medicine, Monash University, Alfred Hospital, Melbourne, VIC 3004
[2]Bone and Joint Research Unit, Service of Rheumatology, IIS-Fundación Jiménez Díaz, Autonomous University of Madrid, Madrid, Spain
[3]Red Temática de Investigación Cooperativa de Envejecimiento y Fragilidad (RETICEF)-Instituto de Salud Carlos III, Madrid, Spain.

Correspondence to:
Raquel Largo, Bone and Joint Research Unit, Service of Rheumatology, IIS-Fundación Jiménez Díaz, Autonomous University of Madrid, Reyes Católicos, 2, 28040, Madrid, Spain. Tel.: 34-91- 550-4978; Fax: 34-91-544-2636; email: rlargo@fjd.es

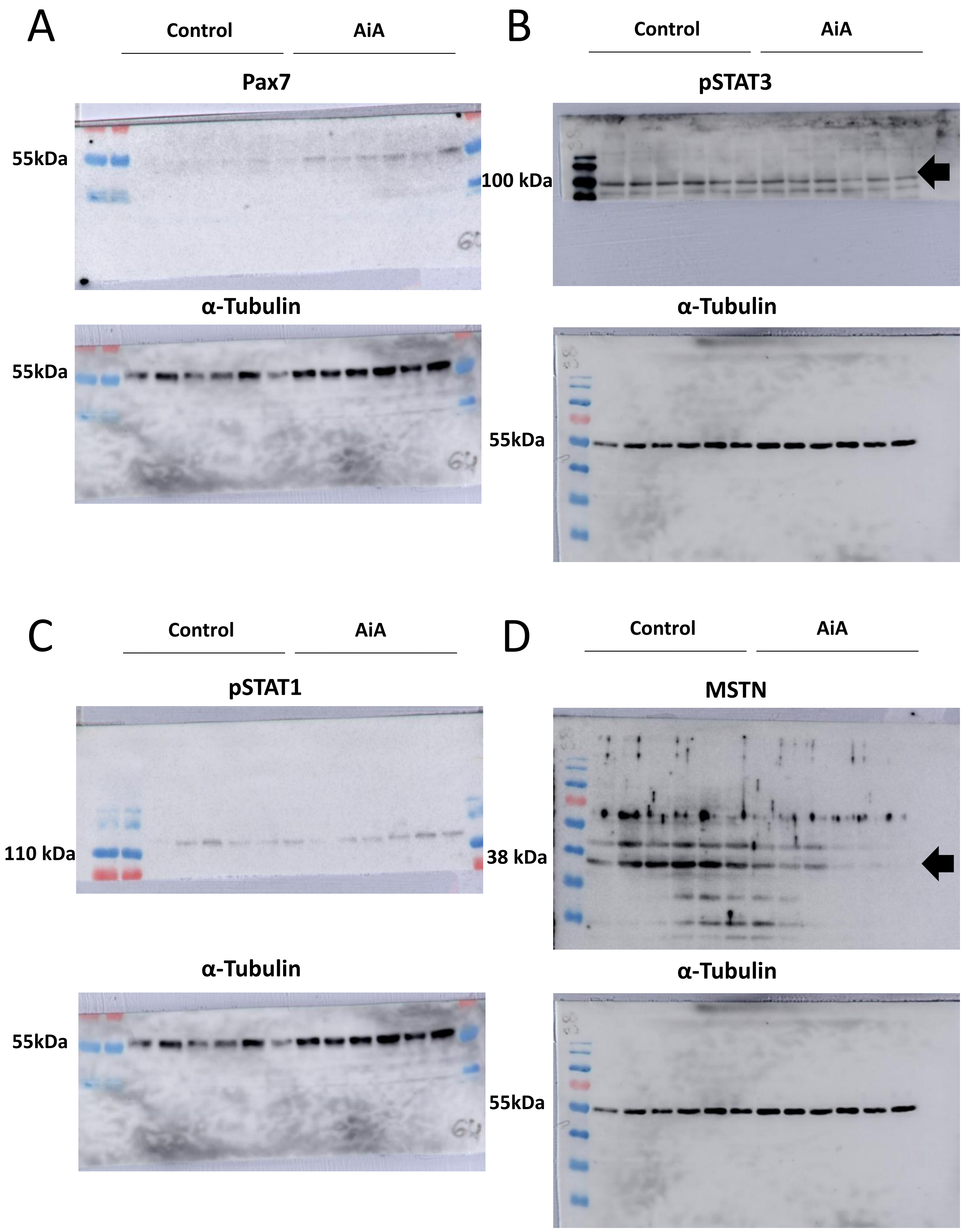

Supplementary figure 1. Full-length blots from gastrocnemius. (A). Pax7 blot of 6 representative animals of each group are shown, control and AiA, respectively. (B). pSTAT3 blot of 6 representative animals of each group are shown, control and AiA, respectively. (C). pSTAT1 blot of 6 representative animals of each group are shown, control and AiA. (D). MSTN blot of 6 representative animals of each group are shown, control and AiA. Normalization of the tubulin blot used the same nitrocellulose membrane for Pax7 and pSTAT1, as well as for pSTAT3 and MSTN (n = 6 rabbits per group). Molecular weights are depicted on the side of the loading control.

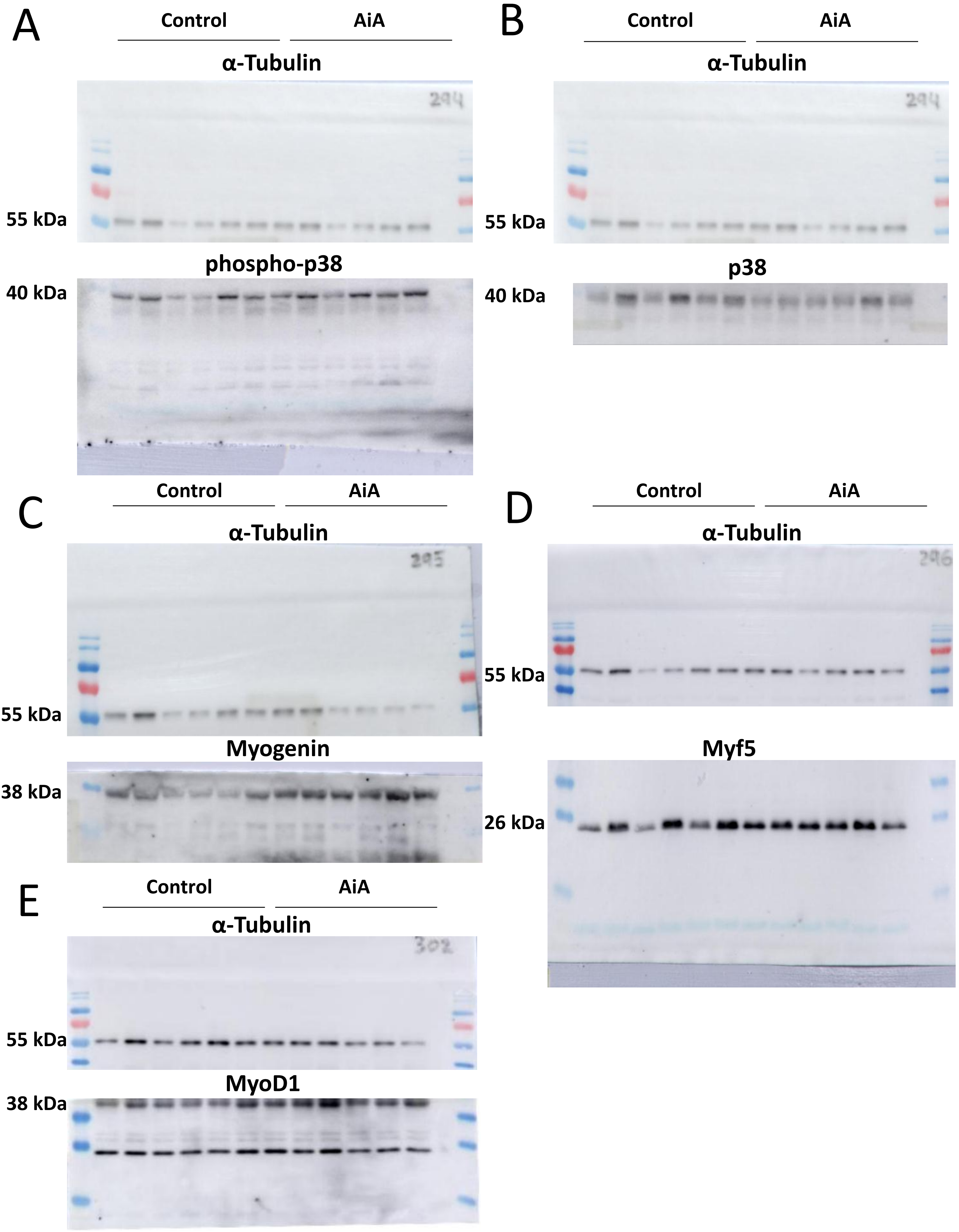

Supplementary figure 2. Full-length blots from gastrocnemius. (A). phosphor-p38 blot of 6 representative animals of each group are shown, control and AiA, respectively. (B). p38 blot of 6 representative animals of each group are shown, control and AiA, respectively. (C). Myogenin blot of 6 representative animals of each group are shown, control and AiA. (D). Myf5 blot of 6 representative animals of each group are shown, control and AiA. (E). MyoD1 blot of 6 representative animals of each group are shown, control and AiA. (n = 6 rabbits per group). Molecular weights are depicted on the side of the loading control.
